# Papillary Thyroid Microcarcinoma: Active Surveillance Against Surgery. Considerations of an Italian Working Group From a Systematic Review

**DOI:** 10.3389/fonc.2022.859461

**Published:** 2022-03-23

**Authors:** Giuseppina Orlando, Gregorio Scerrino, Alessandro Corigliano, Irene Vitale, Roberta Tutino, Stefano Radellini, Francesco Cupido, Giuseppa Graceffa, Gianfranco Cocorullo, Giuseppe Salamone, Giuseppina Melfa

**Affiliations:** ^1^ Department of Surgical Oncology and Oral Sciences, Unit of General and Emergency Surgery, University of Palermo, Palermo, Italy; ^2^ Section of Endocrinology - Department of Health Promotion Sciences Maternal and Infantile Care, Internal Medicine and Medical Specialties (PROMISE), University of Palermo, Palermo, Italy; ^3^ Department of Surgical, Oncological and Oral Sciences, University of Palermo, Palermo, Italy; ^4^ Department of Surgical Oncology and Oral Sciences, Unit of General and Oncology Surgery, University of Palermo, Palermo, Italy

**Keywords:** papillary thyroid microcarcinoma, active surveillance, thyroidectomy, thyroid cancer, quality of life, lymph node metastasis

## Abstract

**Introduction:**

Active surveillance is considered a viable option for papillary thyroid microcarcinoma. Since the last decade of the 20th century, this method has spread from Japan to other countries, but has not yet been fully accepted and validated by the major Western Scientific Societies. In 2016, a systematic review on the results of active surveillance was published, based on two articles that showed encouraging results. Other reviews published subsequently, were mainly based on articles from the Far East. The aim of this review is to assess the most recent results published from 2017 to 2020 on this subject.

**Materials and Methods:**

A systematic literature search was performed on MEDLINE *via* PUBMED, Web of Science, and Scopus according to PRISMA criteria. The MESH terms “papillary thyroid microcarcinoma” and “active surveillance” were adopted. Tumor progression, secondary localizations, and quality of life were the main benchmarks.

**Results:**

Nine studies met the inclusion criteria. The increase in volume ranged from 2.7% and 23.2%; the occurrence of lymph node metastases from 1.3% to 29%; QoL was improved in both articles that addressed this topic. The level of evidence is considered low due to the retrospective and uncontrolled nature of most of the studies included in the review.

**Conclusion:**

The evidence from the literature currently available on AS falls into two strands: a robust data set from the Japanese experience, and an initial experience from Western countries, whose data are still limited but which show a lack of substantial alerts against this practice. Further data is useful to validate the spread of Active Surveillance.

## Introduction

Papillary Thyroid Microcarcinoma (PTmC) is a thyroid cancer measuring 1 centimeter in diameter at most. In most cases, it is diagnosed as an unforeseeable finding after pathology examination of a specimen removed for benign disease in most cases. It is diagnosed less frequently as a suspected infracentimetric thyroid nodule discovered during a routine neck ultrasonography or CT scan, and its presence is rarely revealed after palpable lymph node neck metastases, or in exceptional cases, distant metastases, have been discovered (1). Actually, this tumor is considered the main cause of the increase in the incidence of Papillary Thyroid Carcinoma (PTC) since the widespread use of high resolution ultrasounds has increased the diagnosis of PTmCs (2). Surgery is usually considered the gold standard in treatment of PTmC, although active surveillance (AS) began to take on (3) and has been described in several articles published since this procedure was introduced by Miyauchi at Kuma Hospital in Kobe (Japan) in 1993 and obtained approval by other surgeons (4) until it was adopted at the Cancer Institute Hospital in Tokyo (Japan) in 1995 (5). As a background, epidemiologic data strongly supported this option: in autopsy studies published before 1993, the prevalence of occult PTmCs ranged from 5,7% to 35,6% (1); Takebe (6) showed that the prevalence of PTC in a population undergoing a screening for breast cancer and that was submitted at the same time to a screening for thyroid cancer was 1000-fold the prevalence of clinically evident PTC in the same country and period. These findings led the teams of Kobe and Tokyo to introduce the practice of AS with the aim of identifying cancers growing during observation, considering that the consequent treatment delay did not worsen prognosis and systematic surgery for PTmC has more complications than advantages (5).

In 2016, Alhashemi published the first systematic review on active surveillance for management of low-risk Papillary Thyroid Carcinoma T1 N0 M0. Although the purpose of this article was to evaluate results of AS in all low-risk T1 papillary thyroid carcinomas (less than 2 cm in diameter), all in all it included two articles that evaluated literature concerning AS PTmC (7). Some more recent reviews (8, 9) have emphasised the benefits of this practice, but most of these papers come from Far East countries, often from the same working groups, or groups close in lifestyle and culture.

The aim of this systematic review is to assess the findings of the literature from the period 2016-2020 from an observation point outside the culture and environment in which this practice originated and developed. This is especially so now as a number of Western and European groups have published the first data on their experience with this topic.

## Materials and Methods

This systematic review was performed according to PRISMA criteria (10). According to these guidelines, we selected the studies included in this review as follows.

A systematic literature search was performed on MEDLINE *via* PUBMED, Web of Science and Scopus databases by four independent investigators (AC, IV, GR, and SR) who searched for articles published in English since 2017. We excluded previous papers because we considered Alashemi’s review to be completely exhaustive and in no way improvable, in the reporting period considered (7). The MeSH terms adopted were: papillary thyroid microcarcinoma; active surveillance with Boolean operators AND or OR. We also included all relevant articles cited as references for selected articles and not found with our search. Non-English language articles, reviews, case reports, case series, editorials, and repeated or redundant manuscripts were excluded in advance. In the case of disagreement between investigators on the value of selected papers, a supplementary confrontation was crucial in decision making. Titles of papers were evaluated for the 91 manuscripts retrieved. The abstracts of papers that appeared to be in agreement with the aim of review were read and, if even after this step, the article appeared to be in line with our aims, it was downloaded and read in full.

**Figure 1:**
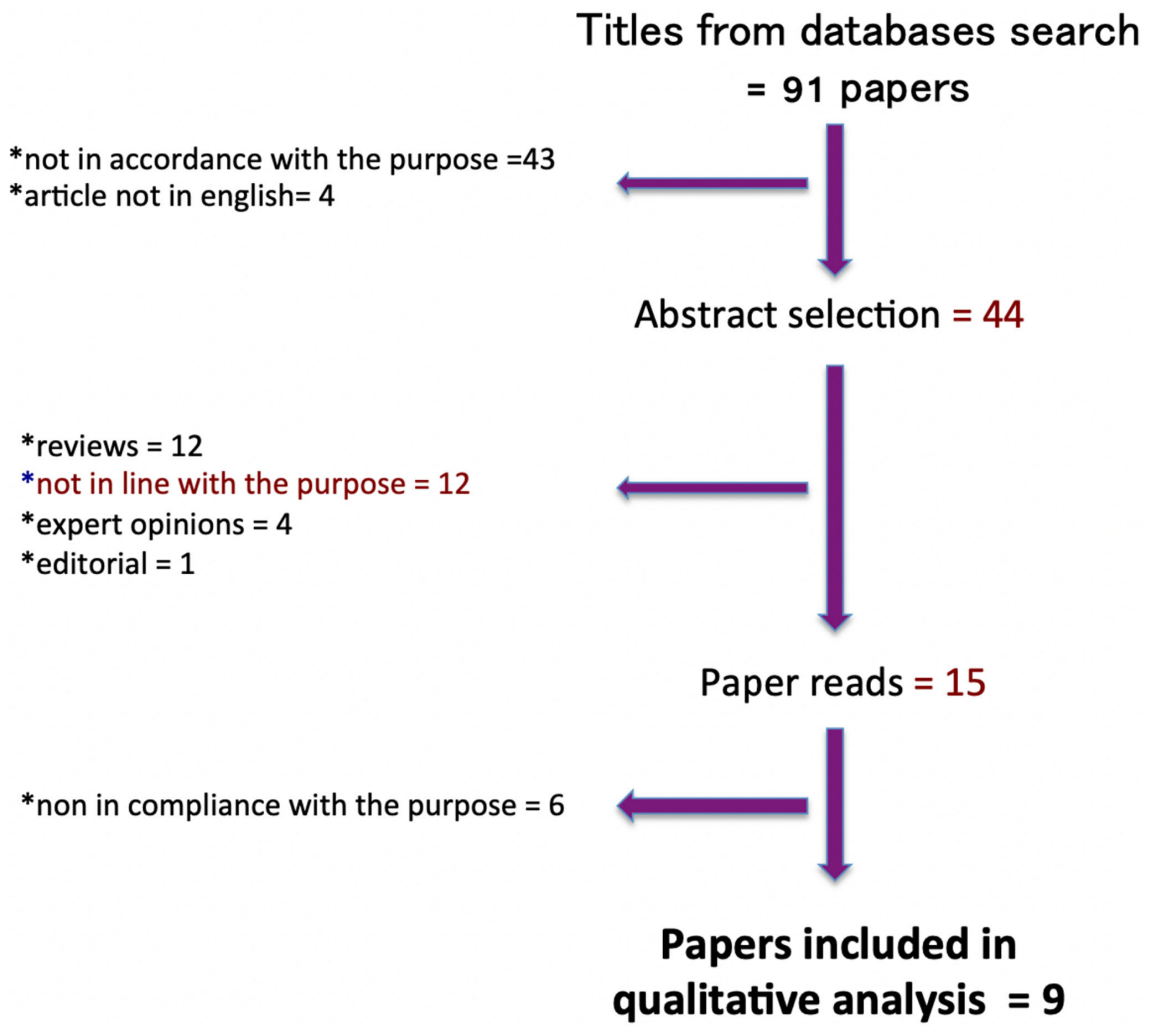
Prisma diagram detailing the literature search process and article selection.

The search process is reported in **Figure 1**.

The main benchmark of the included studies was progression, both in terms of volume increase beyond the parameters of significance (> 3 mm) and in terms of the appearance of secondary localisations. In two studies, of which one was a survey, we also assessed the quality of life (QoL) of patients undergoing active surveillance.

The data obtained did not undergo meta-analysis because they are still considered limited from the point of view of overall numbers, at least for those from Western countries, and in any case, they are not comparable, at the moment, with those already well established from the Kuma Hospital in Kobe and the Tokyo Cancer Institute (4, 5).

## Results

Overall, nine articles met the inclusion criteria for this review.

A retrospective, uncontrolled study by Kwon et al. (11), involving 192 patients with a median follow-up of 31.2 months, aimed at assessing the clinical outcomes of AS, showed an increase in volume in 14% of cases (27 patients). In 12.5% of cases (24 patients), surgical indication was given, 7 of which were due to lymph node metastases.

An uncontrolled retrospective study by Miyauchi et al. (12) of 1211 patients, with a median follow-up of 6.2 years and focusing on the probability of progression, showed an increase in lesion volume for 72 patients, lymph node metastases in 18 patients, and both occurrences in 4 patients.

Kim et al. (13) reported, in an uncontrolled retrospective study of 126 patients, the progression of lesion size in 25, of whom 4 went on to surgery. A further 10 subjects (a total of 14, or 11.1%) were not referred for surgery for another reason. It should be noted that, for the first time, a correlation was found between increased TSH values and progression.

An uncontrolled multicentre study by Oh in 370 patients observed with a median follow-up of 32.5 months and focused on the natural history of the disease, showing 23.2% of volumetric progression, 8.2% of lymph node metastases, and 15.7% of subjects requiring surgery overall due to anxiety caused by the disease condition (14).

Results from the groups in which AS was originally developed have, of course, continued to show encouraging results. The Kuma Hospital group (15) published results from 2288 patients undergoing AS from 2005 to 2017, which showed that disease progression affected 57 patients, while 43 still preferred conversion to surgery during follow-up and 62 patients were operated on during observation on the observer’s indication, either because of the onset of parathyroid disease, or for other reasons. The indication for AS increased significantly after 2011, and a parallel reduction in indications for conversion was observed during the same period.

In recent years, a number of referral working groups have also started practising AS outside the context of South-East Asia and Japan, publishing their first results, especially in the last two years. Rosario (2019) published the first South American study carried out on 77 patients, with a 30-month follow-up. Of these, only 1 patient showed tumour progression, with the appearance of lymph node metastases (16).

A recent study on 93 patients with a follow-up of 19 months (range: 6-54), showed a progression in only 3 of them; however, a further 19 patients withdrew their consent to active surveillance, requiring thyroidectomy. Of these, 9 were operated at the same centre and, among them, 1/9 patients showed minimal extrathyroidal extension and 1/9 a tall cell variant. Ten patients were lost at the follow-up. An increase in volume from 50% to 251% was also observed in 15/93 patients, of whom 2 were among those undergoing conversion from observation to surgery. The remaining 13, despite the substantial increase in volume, are still under observation (17).

Quality of life has been assessed in some studies concerning AS in PTmC. Both agree in the improvement of this parameter in subjects undergoing AS compared to the control group (patients undergoing thyroidectomy). However, it should be highlighted that both studies lack randomisation and have a short follow-up (18, 19).

On the other hand, a survey carried out by Yoshida, from the University of Tokyo, showed that patients’ point of view on AS is strongly influenced by the physician and, conversely, understanding the patient’s expectations is crucial for a shared decision making (20).

**Table 1** is the panel of results of the present research. **Table 2** summarizes the average range of the main outcomes evaluated in the articles included in this review. Concerning the patients scheduled for surgery, we consider it useful to specify that not in all articles it is possible to distinguish the need established by the multidisciplinary team from the patient’s choice. QoL was improved in both articles that addressed this topic.

**Table 1:** Panel of results of systematic review.

Author/Year	Country	Trial	Sackett	Follow-up	Benchmark	Results of active surveillance
Miyauchi et al. 2017 (12)	Japan	Retrospective non-controlled **1211 patients**	IV	6,2 years	disease progression	1) >volume: 72 pts§ **(5,95%)** 2) LN met.*: 18 pts§ **(1,49%)** 1+2: 4 pts§ **(0,33%)**
Kwon et al. 2017 (11)	South Korea	Retrospective non-controlled **192 patients**	IV	31,2 months	Size increase Progression	>volume: 27pts§ **(14,06%)** surgery: 24pts§ **(12,5%)** LN met*: 7/24 **(3,65%)**
Kim et al. 2018 (13)	South Korea	Retrospective non controlled **126 patients**	IV	5 years	correlation TSH/disease progression	progression: 25 pts§ **(19,84%)** surgery: 14 pts§ **(11,1%)** *correlation TSH levels/* *disease progression*
Oh et al. 2018 (14)	South Korea	Retrospective multicentric non-controlled **370 patients**	IV	32,5 months	disease progression	> volume: **23,2%** Surgery: **15,7%** LN met*: **8,2%**
Jeon et al. 2019 (18)	South Korea	Non-randomized 148 @lobectom **43 observed**	III	18-24 months	QoL	*better QoL*
Rosario et al. 2019 (16)	Brazil	Prospective non-randomized **77 observed** 18 surgery	III	30 months	disease progression	1 LN metastasis **(1,3%)**
Molinaro et al. 2020 (17)	Italy	Prospectively collected data non-controlled **93 patients**	IV	19 months	-disease progression -consent discontinuation	progression: 3 pts§ **(3,22%)** discontinuation: 19pts§ **(20,43%)** -9 operated at the same institution **(9,67%)**, of which: *1 minimal extrathyroidal extension *1 tall cell
Yoshida et al. 2020 (20)	Japan	Cross-sectional survey: - **20 pts**§ AS^^ -30 pts§	IV	4,1 years	PTmC related symptoms QoL	reduced state of anxiety improved QoL
Sasaki 2021 (15)	Japan	Surgery Retrospective non-controlled **2288 patients**	IV	2005-2017	disease progression	-progression: 57 pts§ **(2,49%)** -pts§ choice for surgery: 43 **(1,88%)** -need of surgery: 62pts§ **(2,71%)**

The data reported in this table came from articles included in the systematic review.

Sackett: Level of evidence based on Sackett’s scale: I, meta-analysis or large randomized trials (clear cut-off results and low risk for error); II, small randomized trials and moderate/high risk for error; III, non-randomized but prospective with contemporaneous control trials; IV, non-randomized trials with historical controls or retrospective analysis; V, case series without control; expert opinion.

met*, metastasis; Pts§, patients; @lobectom, thyroid lobectomy; QoL, Quality of Life.

AS^^, Active Surveillance.

Bold values = number of patients enrolled for active surveillance.

**Table 2:** Average range of the main outcomes of active surveillance.

OUTCOMES	AVERAGE RANGE
Volume increase/progression	2,49 – 23,2
Lymph node Metastasis (n° of patients)	1,3 – 15,7
Scheduled for surgery	11,1 – 20,43

As it is easy to observe, the overall level of evidence of the systematic review cannot be fully satisfactory, as the quality of the evidence of the individual papers is low, since most of them are uncontrolled, non-randomised, and retrospective studies.

## Discussion

A recent survey published by Sugitani (21) highlights that, at present, more than 50% of low risk PTmCs in Japan are kept under observation (21).

Active surveillance is currently considered, especially in eastern countries, to be a viable option that, in most cases, has a favorable impact on quality of life and costs in the medium to long term (22–24).

On the contrary, small thyroid carcinomas can benefit not only from thyroidectomy, but even from more or less extensive lymph node dissections, depending on their aggressiveness. In order to quantify the number of patients with PTmC requiring enlarged excision, we took into account the large case series published by Lombardi et al. (25), which showed that out of more than 900 PTmC operated on, 9.6% were locally advanced (pT3), 5.6% were N1a and 1.1% N1b (25–27).

Again, a comparison of the data from the Sicilian Thyroid Cancer Registry (SRRTC) with those from a US epidemiological registry (SEER) shows that lymph node metastases are present in PTmC in 27.4% of cases in the former and in 28.9% in the latter; that extrathyroidal extension is present in 7.5% in the former and in 6.2% in the latter; and that, finally, multifocality is observed in 26% in the former and in 33.5% in the latter (28).

It should also be noted that micro pT3 makes up 25% of all pT3, and up 15% of all PTmC. In the light of current knowledge, more aggressive treatment is considered mandatory for these tumors (29, 30).

If these epidemiological data suggest that PTmCs should be assimilated to potentially aggressive forms of thyroid cancer, there are other data that could overturn the judgement: The frequency of incidental microcarcinomas in autopsy case histories is well known (1). Moreover, the results of the screening offered for low-cost thyroid nodular disease to the South Korean population in the early 2000s are well known, which showed a real “surge” in the number of PTC cases detected, with no change in mortality in the subsequent periods (31).

At present, the problem lies in the impossibility of identifying risk criteria for PTmC. In other words, we do not know of any biohumoral, radiological, or genetic indicators that would allow us to identify the limited subset of PTmCs that are destined for neoplastic progression (32).

Promising studies are underway to assess the real clinical impact of molecular tests for the management of thyroid nodules. These studies focus on the analysis of multiple genes and, if the results were to confirm the hypotheses, a complete and extremely high-performance genetic identification could be able to define with much greater accuracy than at present the true nature of samples taken from FNA in patients with thyroid nodules. This could improve the detection of thyroid carcinomas with a more favourable prognosis and therefore candidates for less aggressive treatment or, in selected cases, even for AS. These studies, which from another perspective could redefine the TNM of any thyroid tumour, have not yet had sufficient development in the specific field of PTmC, and therefore can only be considered as a fascinating heuristic hypothesis (33).

These considerations ended up converging with the decades-long experience of Miyauchi et al., published in its evolution since the early nineties and founded on certain theoretical cornerstones, based on the non-variation of prognosis in the case of delayed surgery and the observation that systematic surgery can produce more complications than advantages in terms of prognosis. In this new vision, observation assumes the burden of detecting carcinomas which, as they progress, show potentially more aggressive behavior (4–6).

The prerequisite for the implementation of such a protocol, which substantially modifies the current recommendations in the Western world (ATA 2015), is the systematic bioethical sampling of all 5 mm nodules, in order to purify PTmC from benign nodules. Among the malignant ones, a distinction will be made between “high risk” (lymph node or distant metastases, cytological orientation for high malignancy, suspected invasion of the recurrent), PTmC “not suitable” for AS (protruding from the glandular profile, adherent to the airway or digestive tract with respect to which they determine a right or obtuse dihedral angle) and “suitable,” ideally centroparenchymal, far from the inferior laryngeal nerve, with “reassuring” cytology (5).

The interest aroused by these initial data on this practice has led to a careful evaluation of these results.

The review published by Alashemi in 2016 (7) was carried out after a rigorous selection of 2375 papers, which resulted in only 2 papers being considered suitable. All the remaining literature was based on further reviews, often unsystematic, on data reported by other authors, reiterated or redundant, on unclear reasons and procedures for observation, or other causes of inadequacy. This study selected a number of cases of 1235 (published by Ito) and 322 (published by Sugitani) that allowed us to show a conversion rate to surgery of 15.5% and 8.7%, respectively, on a 5-6.5 year follow up, with a percentage of nodules progressing of about 5% (growth > 3 mm) plus about 1-1.5% presenting lymph node metastases. Despite a mortality rate of 0 in the follow-up period, the author pointed out some weaknesses in the documentation from these papers: there was a lack of clarity about the patients’ reasons for choosing surgery, no data on quality of life, no indicators of disease progression, and finally, no data on TSH suppressive treatment.

The costs were another benchmark that we considered noteworthy, although not consistent with the main aims of this review. A study by Lin was set up on a real group of 349 patients with PTmC recruited from 1985 to 2017 from the institutional registry compared to a hypothetical group of subjects undergoing AS. The study showed that the costs of AS are equivalent to those of surgery after a little more than 16 years, and they are higher thereafter. Moreover, the incidence of permanent complications was also measured in 3.7% of patients undergoing thyroidectomy. This may lead to the conclusion that it is not economically viable to perform AS in younger patients, as they would be destined to a prolonged observation protocol (34).

A recent systematic review developed a cost-effectiveness analysis based on five retrospective trials. This study does not come to any definitive conclusions, but emphasizes the difficulties of carrying out evaluations of this kind, given that the costs evaluated (examinations, ultrasound investigations, surgery, etc.) have an extremely wide variability between the different national reference realities. However, even taking into account these limitations of the study, the authors are inclined to conclude, also in this case, that a younger age could make the cost-effectiveness assessment more favorable for surgery (35).

It should also be noted that AS requires more extensive diagnostic protocols than European standards. In particular, the restrictive indications for FNAB in many European countries conflict with the need to refer a large number of patients with suspicious subcentimetric nodules when adopting a strategy aimed at AS (36).

These considerations are further confirmed by the fact that thyroidectomy can now be performed effectively using minimally invasive techniques (37).

Along with these data, which are undoubtedly discouraging, we should highlight data that strongly supports AS in terms of disease progression and aggressiveness. In a large cohort of patients, drawn from the SEER US database from 1975 to 2015, staged T1-4 N0 M0 and dichotomously stratified between non-surgical (1453) and surgical (54718), Ho et al. (38) showed that there was an overlapping disease-specific survival (DSS), in the two arms of the study, for ‘age < 55 years, for any size of T, while a high statistical significance (p<0.001) was detected only in patients > 75 years with T > 6 cm. Therefore, this study, although retrospective and with populations not specifically divided according to the strict criteria of active surveillance, seems to show that PTC exposes patients to a realistic scale of risk, which increases with tumour size and age (38).

On the other hand, some recent studies, carried out even outside the Far East context, have crossed the 1 cm size threshold to refer patients to AS, demonstrating the feasibility and reliability of this practice even in 1.5 cm diameter tumours and even for any “T” size (39, 40). Nevertheless, it has been reported that tumour size > 1 cm may be associated with a higher rate of lymphovascular invasion (41), so exceeding the original “classical” threshold for AS seems to need further validation studies. Ultimately, it should also be considered that AS should be compared, rather than total thyroidectomy, with haemithyroidectomy, which is itself a low-impact operation for the patient, both in terms of severe complications and resulting quality of life (24). Finally, we consider it useful to stress the importance of the tumor size: in fact, some studies have previously reported that PTMC <5 mm is usually not aggressive, whereas tumors >6 mm present a higher risk of lymph node metastasis (42).

## Conclusion

We conclude that, to date, the scientific literature on AS in PTmC seems to be essentially divided into two different groups: a substantial series of results, mostly from the countries where AS was developed (Kuma Hospital in Kobe and Cancer Institute in Tokyo) and neighbouring sites, and initial, albeit encouraging, European and South American experiences.

The number of patients enrolled in Western trials is still too limited to draw conclusions of possible generalization, but available data allow us to draw some conclusions, which will have to be the subject of more robust and exhaustive evaluations:

-confirmation of PTmC indolence, at least in the vast majority of cases, is an optimal assumption for AS;
-none of the studies published so far, either in the Far East or in Western countries, has shown significant alerts such as to justify a hostile attitude towards AS, which, in the light of these data, could appear preconceived and anti-scientific;
-the studies that are to be published in the near future, and which will be welcome, in addition to progression-related parameters, should investigate the acceptance of AS by patients, taking into account the cultural differences that distinguish Far Eastern countries from Western ones, which are likely to have significant effects on the quality of life.

In any case, it should be taken into account that, in a large proportion of patients who still undergo surgery for PTmC, conservative surgery (haemithyroidectomy) performed with minimally invasive techniques remains a strong argument in favour of surgery.

Further evaluation in a larger scale of patient needs to be performed in order to validate AS in global settings with more robust data.

## Data Availability Statement

The datasets presented in this study can be found in online repositories. The names of the repository/repositories and accession number(s) can be found in the article/supplementary material.

## Author Contributions

Each author made substantial contributions to the work, has approved the submitted version and agrees to be personally accountable for the author’s own contributions and for ensuring that questions related to the accuracy or integrity of any part of the work are appropriately investigated, resolved, and documented in the literature. Conceptualization: GO and GM. Methodology: GSc and SR. Validation: GSa and GG. Formal analysis: GSa. Investigation: AC. Resources: IV. Data curation: FC and AC. Writing: GO. Original draft preparation: AC and IV. Writing – review & editing: GM and GSc. Visualization: SR. Supervision: GM. All authors contributed to the article and approved the submitted version.

## Conflict of Interest

The authors declare that the research was conducted in the absence of any commercial or financial relationships that could be construed as a potential conflict of interest.

## Publisher’s Note

All claims expressed in this article are solely those of the authors and do not necessarily represent those of their affiliated organizations, or those of the publisher, the editors and the reviewers. Any product that may be evaluated in this article, or claim that may be made by its manufacturer, is not guaranteed or endorsed by the publisher.
